# Clinical Experience with Chloral

**Published:** 1870-03

**Authors:** 


### Miscellaneous.

### Clinical Experience with Chloral.

TRANSLATED BY ROBERT AMORY, M. D.

Having come across the review by Dr. Bricheteau of the experiments on chloral made by Messrs. Liebreich, Dumas, Richardson, Spencer Wells, Dumarquay and Follet, Dieulafoy and Krishaber, Labbe, Personne, as well as some results of clinical experience by the reviewer, I translate his conclusions for the benefit of your readers, who may not have seen this article, in the *Bull. Gen. de Ther.*, Nov. 30th.

1st. Hydrated chloral, or chloral hydrate, is a powerful sedative to the nervous system, both motory and sensory.

2d. If the hydrate of chloral is not crystalline and very pure, so that in the evolution of chloroform by the addition of potass, there is no brownish color produced in the residuum, it is either without action or may be very dangerous.

3d. A larger dose than 75–90 grains (5–6 grammes) should not be given at any one time to an adult, and to a child the commencing dose should be 15–30 grains.

4th. The preparations of chloral ought not to be made too long before the administration, for they lose their character and become inefficacious.

5th. The administration may be made by the mouth. Baths produce the same effect as when the stomach is used, but the latter is the better method.

6th. Its use should not be employed in patients who have organic disease of the heart or brain.

7th. It is on account of the production of chloroform in the blood, from its alkaline reaction, that the ingested chloral produces sleep and anæsthesia.

8th. It is dangerous in man to employ its hypodermic injection.

9th. The arterial tension is augmented by the influence of chloral, at the same time that a diminution in the frequency of the pulse is produced; this tension diminishes on awaking, as the sphygmographic trace indicates.

10th. The urine during the sleep produced by chloral is neutral; and bailed with Fehling's liquid, it does not reduce at first the copper salts; but the next day it becomes, when the chloral has passed through the kidneys, more dense, and will cause the reduction of the copper salts; this may cause a belief in the presence of sugar in the urine which does not in point of fact exist.

11th. Hydrate of chloral rarely causes vomiting, and never purges.

12th. The temperature is slightly lowered, by doses which are not toxical, which points to its being an algid medicament.

13th. By the hydrate of chloral the cutaneous perspiration is diminished and the skin becomes dryer than in the normal state

14th. This drug can be precisely dosed for the efficacious production of anæsthesia ; whilst in the inhalations of chloroform for the same purpose, the vapor cannot so be dosed. The uncertain use of chloroform vapor makes its use very dangerous.

15th. The action of hydrate of chloral is exactly that of chloroform, but its production is slower, and its duration longer if the chloral is used.

16th. In some patients submitted to the action of chloral there is a muscular and moral excitement similar to alcohol drunkenness; but this intoxication is not disgusting nor disagreeable.

17th. In almost all there is a sleep, remarkable from a very pronounced anæsthesia, and rarely accompanied with hyperæsthesia.

18th. The anæsthesia is proportioned to the dose employed, and in the dose of 30 to 75 grains, according to the age, it is complete, and the cautery of Vienna paste may be used, or even teeth extracted, without causing pain.

19th. Compared with opium, which often causes vomiting, which destroys the appetite, which stimulates, heats, constipates, excites the cutaneous transpiration, produces slowly a heavy sleep, and leaves after awaking a *malaise* from prolonged sleep; hydrate of chloral does not produce vomiting, does not constipate, nor destroy the appetite; it dries and may even cool the skin; it quickly causes a light sleep which lasts some time; finally, on awaking, it leaves no heaviness of spirits nor feelings of somnolence, and can be taken several days in succession.

20th. In a large dose, this drug produces algidity, whilst opium produces heat and diaphoresis.

21st. A dose of 45 to 75 grains can be repeated two or three times a day without inconvenience, and there results two or three times several hours of sleep, separated by a short interval of wakefulness.

22d. As a therapeutic agent, the chloral is sedative to violent pains in gout, to atrocious sufferings from nephritic colic or from dental caries, or from burns. In other words, it is the prime anæsthesia administered by the stomach.

23d. In the cases where a resort to chloroform is made, the chloral can be used to appease the pangs of natural parturition, to facilitate obstetrical operations and to combat puerperal eclampsia.

24th. Finally, it is the most prompt and efficacious remedy to employ in intense chorea, when we wish to cause a rapid cessation to an agitation which of itself threatens the life of the patient.

Why will not some of your readers who have a large obstetrical practice, test its benefit in parturition ?—*Boston Medical and Surgical Journal.*

